# Genetic Polymorphism of Angiotensin Converting Enzyme and Risk of Coronary Restenosis after Percutaneous Transluminal Coronary Angioplasties: Evidence from 33 Cohort Studies

**DOI:** 10.1371/journal.pone.0075285

**Published:** 2013-09-30

**Authors:** Shen Wang, Yuxiang Dai, Lingling Chen, Zhibing Dong, Yunpeng Chen, Chenguang Li, Xin Zhong, Wenhui Lin, Jifu Zhang

**Affiliations:** 1 Department of Cardiology, The first people’s hospital of Wenling, Wenling, Zhejiang, People’s Republic of China; 2 Shanghai Institute of Cardiovascular Diseases, Zhongshan Hospital, Fudan University, Shanghai, People’s Republic of China; University of Tampere, Finland

## Abstract

**Background:**

In the past decade, a number of cohort studies studies have been carried out to investigate the relationship between the insertion/deletion polymorphism of the gene encoding angiotensin-converting enzyme and risk of restenosis after percutaneous transluminal coronary angioplasties in patients. However, these studies have yielded contradictory results. Genetic association studies addressing this issue are frequently hampered by insufficient power. We therefore performed a meta-analysis of the published studies to clarify this inconsistency and to establish a comprehensive picture of the relationship between ACE I/D polymorphism and post-PTCA restenosis risk.

**Methods:**

Databases including Pubmed, EMBASE, ISI Web of Science, EBSCO, Cochrane Library databases and CNKI were searched to find relevant studies. Odds ratios (ORs) with 95% confidence intervals (CIs) were used to assess the strength of association. The random-effects model was applied, addressing heterogeneity and publication bias.

**Results:**

A total of 33 cohort studies involving 11,099 subjects were included. In a combined analysis, the OR for post-PTCA restenosis of the ACE DD genotype was 1.61 (95% CI: 1.27–2.04; *P*<10^−5^). In the subgroup analysis by intervention, significantly increased risks were also found in PTCA-stent and PTCA-balloon for the DD genotype of the polymorphism.

**Conclusions:**

Our meta-analysis showed that the DD genotype of ACE I/D polymorphism was significantly associated with increased risk of restenosis, particularly for PTCA-stent.

## Introduction

Coronary artery disease (CAD), including its most severe complication, myocardial infarction (MI), is the leading cause of morbidity and mortality worldwide. Percutaneous transluminal coronary angioplasties (PTCA) is now a well established treatment for widening the lumen of coronary arteries stenosed by atherosclerotic lesions. The main limitation of PTCA is restenosis in 30–40% of patients, typically occurring between 1–3 months [1], [2]. A number of clinical and angiographic variables, including advanced age, diabetes mellitus, hyperlipidaemia, hypertension, unstable angina, severe coronary artery stenosis and long lesions, have been reported to be associated with an increased risk of restenosis after PTCA [3]–[6]. However, only 30% of restenosis could be predicted from clinical and angiographic variables [5]. The hypothesis of a genetic susceptibility to explain the 30% to 40% of patients affected by restenosis has been raised.

Inappropriate activation of the renin–angiotensin system may play a part in the development of many cardiovascular disorders [7], [8]. Experimental studies favor the major role of the renin angiotensin system (RAS) in vessel healing after PTCA [9]–[11]. A common insertion/deletion polymorphism within the angiotensin-I converting enzyme gene (ACE-I/D) has been reliably associated with substantial differences in the plasma and tissue angiotensin-converting enzyme (ACE) activity in a codominant fashion not only in persons of European descent, but also in other populations such as Hispanics [12]–[14]. Individuals carrying the D allele have higher ACE activity, which has been proposed as an intermediate phenotype of potential relevance for the development of high blood pressure and subclinical atheroma (i.e., higher intima-media thickness of the carotid artery) [13], [15].

It has been suggested that the incidence of coronary restenosis after a percutaneous coronary intervention is much higher in patients with the angiotensin converting enzyme DD genotype (which is associated with particularly high plasma angiotensin converting enzyme levels) than in others. However, these studies have yielded apparently conflicting results. These disparate findings may be partly due to insufficient power, false-positive results, and publication biases. The interpretation of these studies has been further complicated by the use of different populations. We therefore performed a meta-analysis of the published studies to clarify this inconsistency and to establish a comprehensive picture of the relationship between ACE I/D polymorphisms and post-PTCA restenosis risk.

## Materials and Methods

### Literature Search Strategy

Electronic databases (Pubmed, EMBASE, ISI Web of Science, EBSCO, Cochrane Library databases and CNKI) were searched up to March 2013 for all genetic association studies evaluating the ACE-I/D polymorphism and coronary restenosis after percutaneous transluminal coronary angioplasty (PTCA) in humans in all languages. The search strategy contained both medical subject heading terms and text words as follows: “angiotensin-converting enzyme” or “ACE” or “peptidyl-dipeptidase A,” in combination with “angioplasty” or “stent” or “balloon” or “stenting” or “percutaneous” or “PTCA,” and combined with “genetic” or “polymorphism(s)” or “variations(s)” or “genotype” or “gene(s)”.

### Eligible Studies and Data Extraction

Eligible studies had to meet all of the following criteria: (1) original papers containing independent data which have been published in peer-reviewed journal, (2) genotype distribution information or odds ratio (OR) with its 95% confidence interval (CI) and P-value, (3) restenosis had to be defined as >50% luminal diameter stenosis at follow-up angiography after an initially successful angioplasty procedure, and (4) prospective cohort studies or case–control studies.

The following information was extracted independently and entered into separate databases by two authors from each qualified study: first author’s surname, publication year, ethnicity, genotyping method, definition of restenosis, frequency of insertion or deletion genotypes, sex, mean age, and duration of follow up. The results were compared, and disagreements were discussed among all authors and resolved with consensus.

### Statistical Methods

We estimated ORs by comparing cases who developed coronary restenosis after a percutaneous coronary intervention with controls who did not within the same study. The per-allele OR of the D allele was compared between cases and controls. Then, we estimated the risks of the heterozygous and homozygous genotypes on restenosis compared with the wild-type homozygous. Cochran’s Q-statistic test was performed to assess possible heterogeneity in the combined studies [16]. Random-effects and fixed-effect summary measures were calculated as inverse variance-weighted average of the log OR [17], [18]. The results of random-effects summary were reported in the text because it takes into account the variation between studies. Sources of between-study heterogeneity were explored using random effect meta-regression models with restricted maximum likelihood estimation. The prespecified characteristics for assessment of sources of inter-study heterogeneity were: ethnicity and study size (number of cases: ≥300, or <300). Ethnic group was defined as Caucasian (i.e., people of European origin), East Asian (e.g., Chinese, Japanese, and Korean), and Others. One-way sensitivity analysis was performed to assess the stability of the results, namely, a single study in the meta-analysis was deleted each time to reflect the influence of the individual data set to the pooled OR. Funnel plots and the Egger’s test were used to examine the influence of publication bias (linear regression analysis) [19]. All P values are two-sided at the P = 0.05 level. All of the statistical tests used in this meta-analysis were performed by STATA version 10.0 (Stata Corporation, College Station, TX).

## Results

### Study Characteristics

**Figure 1:**
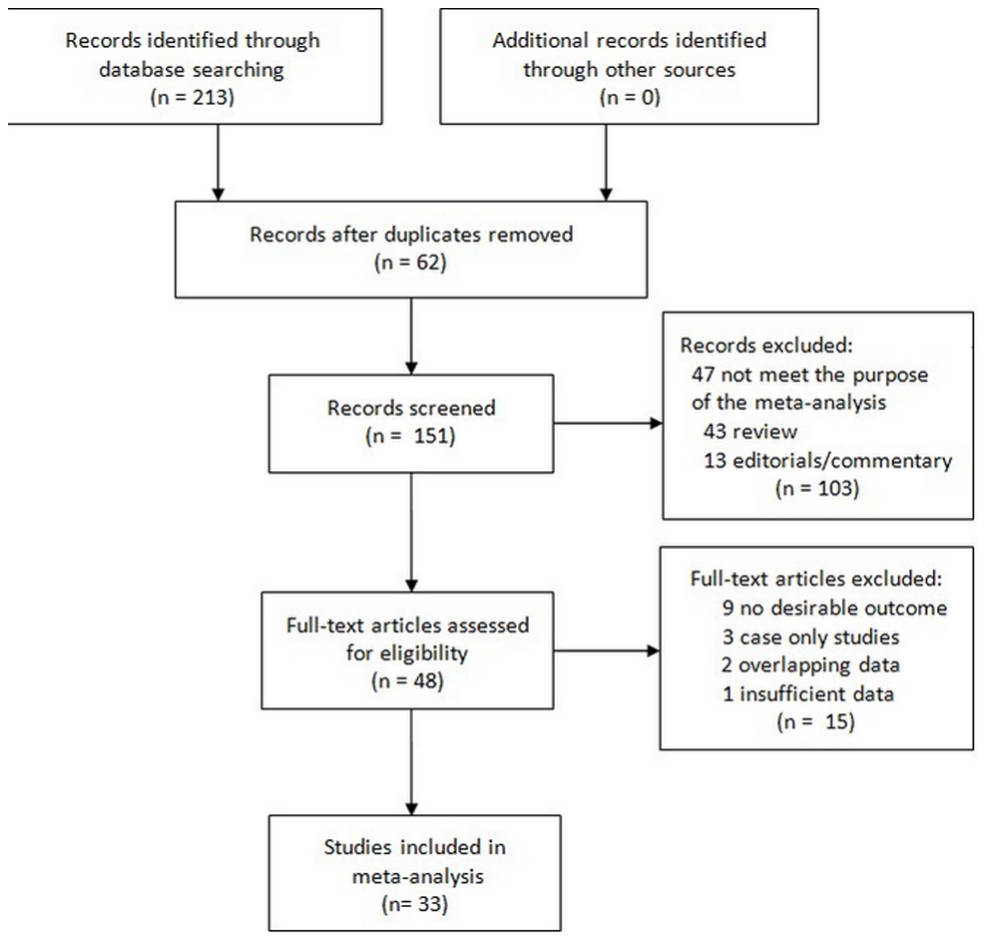
Study selection process.

The combined search yielded 213 references. Study selection process was shown in Figure 1. Overall, 33 studies with 11,099 patients investigating restenosis were included [20]–[52]. 11 studies used PTCA with balloon angioplasty alone (PTCA-balloon), 22 studies used PTCA with bare-metal stent deployment (PTCA-stent). Of the patients, 77.8% were Caucasians, 19.5% were East Asians, and 2.7% were other ethnic origins. The detailed characteristics of the studies included in this meta-analysis are shown in Table S1.

### Meta-analysis Results

**Figure 2:**
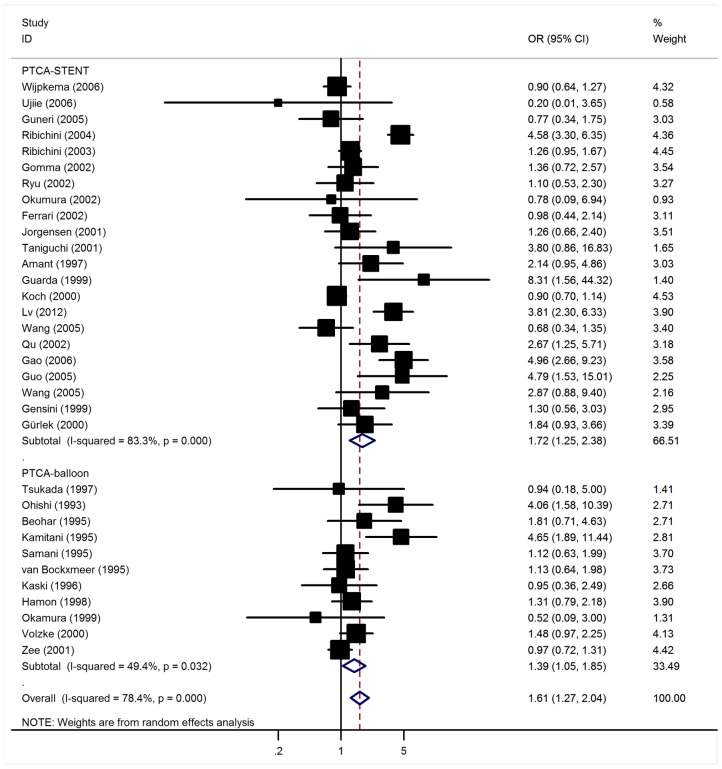
Forest plot for association between DD homozygous of ACE I/D polymorphism and coronary restenosis risk.

Significant association between the ACE I/D polymorphism and restenosis risk were found. Overall, the combined OR for restenosis in patients with the DD genotype was 1.61 [95% CI: 1.27–2.04, P(Z)<10^−5^, P(Q) <10^−5^; Figure 2]. Since the biological phenomena underlying restenosis after PTCA-balloon and PTCA-stent are distinct (negative remodeling due to elastic recoil versus neointimal hyperplasia and inflammatory response, respectively) [53], separate meta-analyses for PTCA-balloon and PTCA-stent were performed.

### Association between ACE I/D Polymorphism and Restenosis Risk after PTCA-stent

In the overall analysis, the ACE I/D polymorphism was significantly associated with elevated restenosis risk with a per-allele OR of 1.37 (95% CI: 1.09–1.71, P = 0.006). Significant associations were also found for DD genotype with OR of 1.72 (95% CI: 1.25–2.38, P = 0.001, Figure 2); while no association was detected for ID genotype (OR = 1.09, 95% CI: 0.87–1.36).

In the stratified analysis by ethnicity, significant risks were found among East Asians for DD genotype (OR = 2.19, 95% CI: 1.2–3.80, P = 0.005). However, no significant association was found for Caucasians and other ethnic populations in all genetic models (Table 1). When studies were grouped by size, however, the combined OR for restenosis were 1.55 (95 CI: 1.12–2.16, P = 0.009) for the 15 studies with less than 300 cases, and 1.93 (1.09–3.41, P0.03) for the 7 studies with more than 300 cases.

**Table 1:** Results of meta-analysis for ACE I/D polymorphism and restenosis risk.

Intervention	Total/subgroup analysis	No. of studies	D allele			ID heterozygous			DD homozygous		
			OR (95%CI)	P(Z)	P(Q)	OR (95%CI)	P(Z)	P(Q)	OR (95%CI)	P(Z)	P(Q)
PTCA-stent	Total	22	1.37 (1.09–1.71)	0.006	<10^−5^	1.09 (0.87–1.36)	0.45	0.01	1.72 (1.25–2.38)	0.001	<10^−5^
	East Asian	10	1.45 (0.93–2.27)	0.10	<10^−4^	1.27 (0.81–1.97)	0.29	0.01	2.19 (1.26–3.80)	0.005	<10^−4^
	Caucasian	9	1.20 (0.90–1.61)	0.21	<10^−4^	0.98 (0.83–1.16)	0.83	0.39	1.41 (0.91–2.18)	0.12	<10^−4^
	Other	3	1.67 (0.89–3.13)	0.11	0.05	2.42 (0.99–5.39)	0.06	0.52	1.84 (0.65–5.19)	0.25	0.03
	No. of cases <300	7	1.20 (0.95–1.65)	0.02	<10^−4^	1.03 (0.75–1.42)	0.85	0.10	1.55 (1.12–2.16)	0.009	0.03
	No. of cases ≥300	15	1.58 (1.08–2.33)	0.11	<10^−4^	1.17 (0.85–1.59)	0.33	0.01	1.93 (1.09–3.41)	0.03	<10^−4^
PTCA-balloon	Total	11	1.25 (0.96–1.64)	0.10	<10^−5^	1.04 (0.60–1.80)	0.89	<10^−4^	1.39 (1.05–1.85)	0.02	0.03
	East Asian	4	1.80 (0.81–3.98)	0.15	0.003	1.23 (0.78–1.94)	0.37	0.79	2.24 (0.87–5.76)	0.09	0.07
	Caucasian	7	1.06 (0.85–1.32)	0.60	0.02	0.85 (0.40–1.80)	0.68	<10^−4^	1.16 (0.96–1.40)	0.12	0.69
	No. of cases <300	9	1.31 (0.88–1.94)	0.18	<10^−5^	0.93 (0.46–1.87)	0.84	<10^−4^	1.52 (1.03–2.23)	0.03	0.05
	No. of cases ≥300	2	1.16 (0.86–1.57)	0.33	0.07	1.24 (0.90–1.71)	0.19	0.30	1.17 (0.78–1.75)	0.45	0.11
Overall	/	33	1.33 (1.12–1.59)	0.001	<10^−5^	1.06 (0.85–1.31)	0.60	<10^−4^	1.61 (1.27–2.04)	<10^−5^	<10^−4^

The data on genotypes of the ACE I/D polymorphism among cases stratified by use of ACE inhibitors were available in 5 studies (including 422 restenosis cases and 1363 controls). Patients on ACE inhibitor treatment with the DD genotype of the ACE gene had a significantly higher in-stent restenosis rate than those without this treatment with an OR of 1.59 [95% CI: 1.06–2.38, P(Z) = 0.02, P(Q) = 0.29] and 1.09 [95% CI: 0.81–1.45, P(Z) = 0.58, P(Q) = 0.87], respectively.

Significant heterogeneity was present among the 22 studies (P<0.05). In meta-regression analysis, follow up (P = 0.07), mean age of cases (P = 0.11), sex distribution (P = 0.19), did not significantly explained such heterogeneity. By contrast, ethnicity (P = 0.02) and sample size (P = 0.007) were significantly correlated with the magnitude of the genetic effect.

### Association between ACE I/D Polymorphism and Restenosis Risk after PTCA-balloon

Overall, the per-allele OR of I/D polymorphism of ACE for restenosis risk after PTCA-balloon was 1.25 (95% CI: 0.96–1.64, P = 0.10), with corresponding results for ID heterozygous and DD homozygous of 1.04 (95% CI: 0.60–1.80, P = 0.89) and 1.39 (95% CI: 1.05–1.85, P = 0.02, Figure 2), respectively. However, there was significant heterogeneity among included studies (P<0.05). Sample size (P = 0.02) explained a large part of the heterogeneity, whereas ethnic group (P = 0.16), follow up duration (P = 0.23), mean age of cases (P = 0.73), and sex distribution among cases (P = 0.38) explained little heterogeneity. When studies were stratified for ethnicity, no significant risks were found for Caucasians and East Asians in all genetic models (Table 1). By considering sample size subgroups, the OR was 1.52 (95% CI: 1.03–2.23, P = 0.03) in larger studies compared to 1.17 (95% CI: 0.78–1.75, P = 0.45) in relative small studies.

### Sensitivity Analyses and Publication Bias

**Figure 3:**
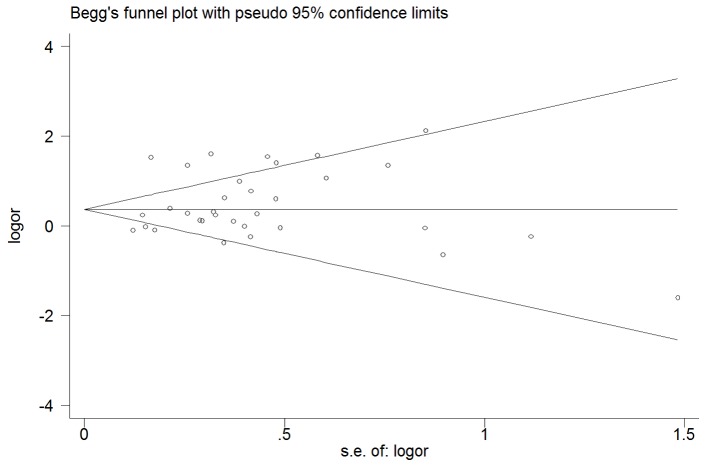
Begg’s funnel plot for DD of I/D polymorphism of ACE and restenosis risk.

In order to assess the stability of the results of the meta-analysis, we performed a sensitivity analysis through sequentially excluded individual studies. Statistically similar results were obtained after sequentially excluding each study, suggesting stability of the meta-analyses. Begg’s funnel plot and Egger’s test were performed to evaluate the publication bias of literatures. As shown in Figures 3, the shape of the funnel plots seemed symmetrical for the polymorphism, suggesting no publication bias among the studies included. The statistical results still did not show publication bias (P = 0.26, Figure S1).

## Discussion

Restenosis after percutaneous coronary interventions has represented a large clinical problem since the introduction of balloon angioplasty, and it remains the major drawback of this technique even after the introduction of stent implantation [1], [54], [55]. There have been various attempts to identify predictors of post-PTCA restenosis on the basis of patient-related, angiographic or procedural factors [4]–[6]. The initial report of an association of the ACE I/D polymorphism and restenosis after angioplasty was based on a rather small sample of only 82 Japanese patients [20]. These findings were not reconfirmed by subsequent, somewhat larger studies [22], [25], [29], although others reported results more in line with the original publication [21], [23], [24]. Because of the limited size of all these studies, the actual contribution, if any, of the ACE I/D marker to the risk to developing post-PTCA restenosis remain difficult to assess. This is the most comprehensive meta-analysis examined the ACE I/D polymorphism and the relationship to susceptibility for post-PTCA restenosis. Its strength was based on the accumulation of published data giving greater information to detect significant differences. In total, the meta-analysis involved 33 studies for restenosis which provided 11,099 subjects.

The combined evidence suggested that ACE DD genotype did contribute to the development of post-PTCA restenosis. Significant associations were found in restenosis risk after coronary stenting; while marginal significant associations were observed in restenosis risk after balloon PTCA. The mechanisms responsible for restenosis after conventional balloon angioplasty and stent implantation are different. Restenosis after conventional balloon angioplasty is caused not only by activation of the RAS, but also by complex factors including elastic recoil, platelet deposition, and thrombus formation [56], [57]. Restenosis after stent implantation, however, is predominantly associated with activation of the RAS [58]. ACE may play a key role by inducing in-stent cell growth secondary to the production of angiotensin II and inhibition of bradykinin [59].

In humans, the level of plasma ACE is stable in an individual but is highly variable between individuals [12]. It has been shown that ACE activity in plasma and cardiac tissue is generally regulated by ACE polymorphism and increases in patients with the DD genotype [12], [60]. This increased activity of ACE may account for the higher degree of neointimal thickening observed in D allele bearers. Bonithon-Kopp et al reported that high plasma concentrations of ACE were associated with a statistically significant increase of common carotid artery intima-media thickening [61]. Another study reported an association between the DD genotype and the extent of common carotid artery intima-media thickening [62]. These studies suggested that long-term exposure to high levels of plasma ACE may be involved in structural changes in the arterial wall. Evidence from animal models suggests that the renin-angiotensin system is involved in the vascular response to balloon injury. ACE has been shown to be induced in such lesions and angiotensin II promotes smooth-muscle cell proliferation in the injured wall [9]. ACE inhibitors and more recently angiotensin II receptor antagonists have been shown to significantly reduce the size of the neointima [63], [64].

Despite positive experimental data, randomised studies in human beings have, so far, shown no benefit of ACE inhibition in the prevention of restenosis after PTCA [65]. In our subgroup analysis of patients on ACE inhibitors, an increased rate of in-stent restenosis was confined to the DD genotype group suggesting a protective role of the I-allele. However, this kind of post hoc analysis should be evaluated with extreme caution, and further studies are needed to evaluate this issue. On the other hand, a randomized study by Okamura et al. [31] demonstrated that DD genotype patients treated with imidapril (an ACE inhibitor) compared with control subjects had a higher restenosis rate after balloon angioplasty. In addition, a recent randomized, double-blind, placebo-controlled study by Meurice et al. [66] of 91 DD genotype patients treated with the ACE inhibitor quinapril reported an increase in late lumen loss in the quinapril-treated patients.

In meta-analysis, heterogeneity evaluation was always conducted in statistical analysis. Thus, several subgroup meta-analyses were performed according to ethnicity and sample size. However, significant between-study heterogeneity was observed in most comparisons. First, ethnic differences may contribute to these different results, since the D allele distributions of the ACE I/D polymorphism varies between East Asian and Caucasian populations, with a prevalence of ∼40%, and ∼55%, respectively. Second, this conflicting association could also be explained by study design or small sample size. On the other hand, since post-PTCA restenosis is a complex process, the effect of single genetic factor on the risk of restenosis may be more pronounced in the presence of other common genetic or environmental cardiovascular risk factors leading to arterial damage through compromised endothelium (i.e. hypertension, smoking, obesity, hypercholesterolemia, diabetes).

In interpreting the results, some limitations of this meta-analysis should be addressed. Firstly, our results were based on unadjusted estimates, while a more precise analysis should be conducted if all individual raw data were available, which would allow for the adjustment by other co-variants including age, ACE inhibitors using, drinking status, cigarette consumption, and other lifestyle. Secondly, in the subgroup analysis by ethnicity, the number of studies and subjects analyzed was small, and the statistical power was so low that caution should be taken in interpreting these results. Thirdly, although the funnel plot and Egger’s test showed no publication bias and although an exhaustive literature search was done, it is likely that some publications and unpublished data were overlooked. Selection bias for the meta-analysis might have occurred.

To conclude, our meta-analysis showed that the DD genotype of ACE I/D polymorphism was significantly associated with increased risk of restenosis, particularly for PTCA-stent. It is also known that the pathogenesis of post-PTCA restenosis is complex and polygenetic in the vast majority of patients, with several genes, each with a small to moderate effect, acting individually, together or in association with important environmental determinants. Larger studies of different ethnic populations, especially with detailed individual information, are needed to confirm our findings.

## Supporting Information

Figure S1 **Test publication bias on studies of the DD of I/D polymorphism of ACE and restenosis using Egger test.**
(TIF)

Table S1 Characteristics of studies included in a meta-analysis of the association between ACE I/D polymorphism and restenosis risk.
(DOCX)

Checklist S1 (DOC)
