# Supplementary figures and images for: Genetic Polymorphism of Angiotensin Converting Enzyme and Risk of Coronary Restenosis after Percutaneous Transluminal Coronary Angioplasties: Evidence from 33 Cohort Studies
Source: PLoS One. 2013 Sep 30;8(9):e75285. doi: 10.1371/journal.pone.0075285 (PMC3787085; doi:10.1371/journal.pone.0075285)

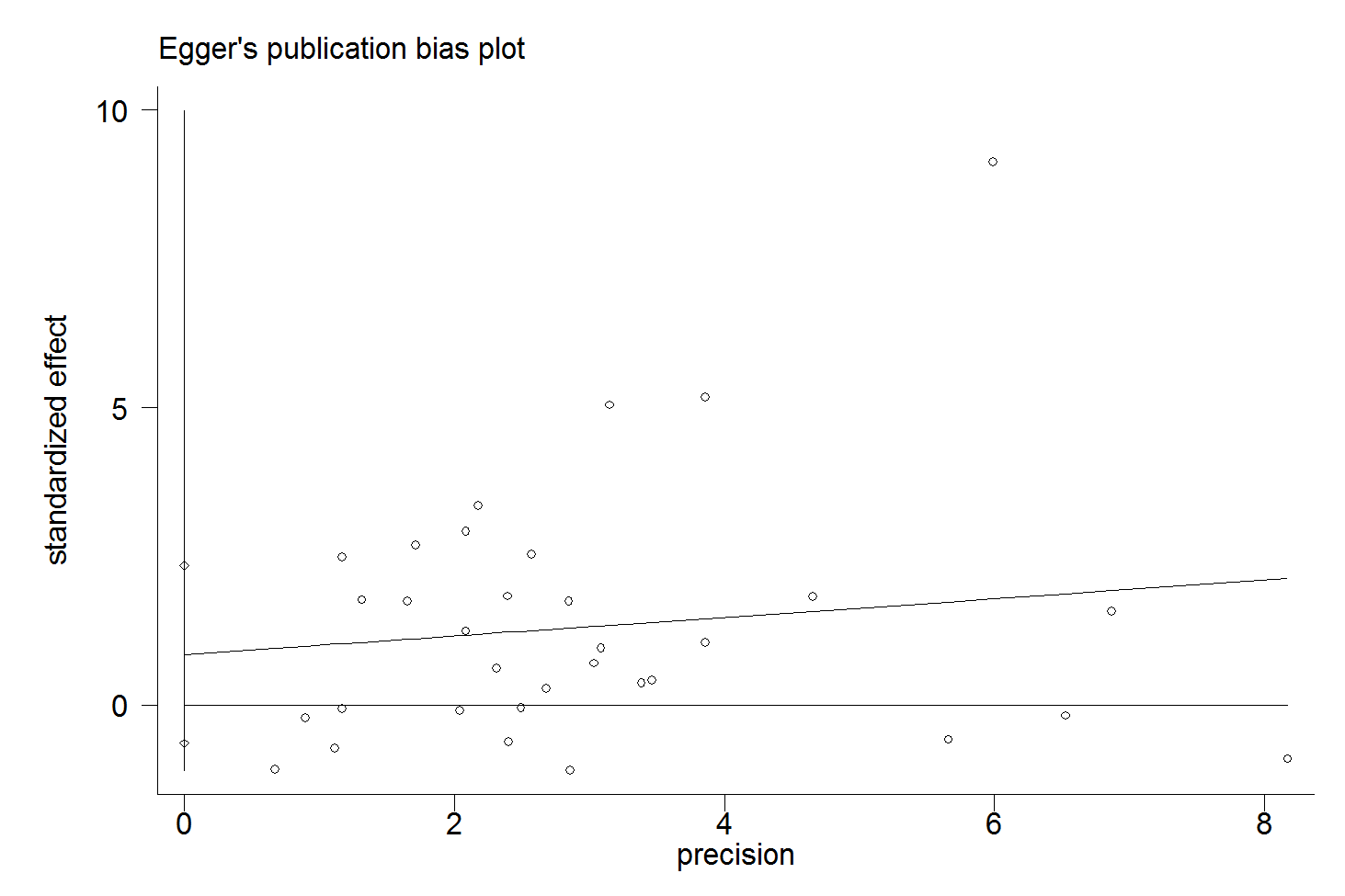

Supplement: Figure S1 — Test publication bias on studies of the DD of I/D polymorphism of ACE and restenosis using Egger test. (TIF) [file pone.0075285.s001.tif]
