# Supplementary material for: Genetic Polymorphism of Angiotensin Converting Enzyme and Risk of Coronary Restenosis after Percutaneous Transluminal Coronary Angioplasties: Evidence from 33 Cohort Studies
Source: PLoS One. 2013 Sep 30;8(9):e75285. doi: 10.1371/journal.pone.0075285 (PMC3787085; doi:10.1371/journal.pone.0075285)
Supplement: Table S1 — Characteristics of studies included in a meta-analysis of the association between ACE I/D polymorphism and restenosis risk. (DOCX) [file pone.0075285.s002.docx]

**Table S1.** Characteristics of studies included in a meta-analysis of the association between ACE I/D polymorphism and restenosis risk

| Study | Year | Ethnicity | Study design, FU | Intervention | Restenosis definition | No. of cases/controls | Mean age | Sex (male %) |
| --- | --- | --- | --- | --- | --- | --- | --- | --- |
| Ohishi [20] | 1993 | Japanese | Cohort, 6 months | PTCA-balloon | diameter stenosis >50% | 32/50 | NA | NA |
| Beohar [21] | 1995 | American | Cohort, 3 months | PTCA-balloon | diameter stenosis ≥50% | 64/25 | 63.9 | NA |
| Samani [22] | 1995 | British | Cohort, 4 months | PTCA-stent | diameter stenosis ≥50% | 110/123 | NA | 83.3 |
| Kamitani [23] | 1995 | Japanese | Cohort, 6 months | PTCA-balloon | diameter stenosis >50% | 38/52 | 52.0 | 100 |
| van Bockxmeer [24] | 1995 | Australian | Cohort, 6 months | PTCA-balloon | diameter stenosis >50% | 88/119 | 57.0 | 82.1 |
| Kaski [25] | 1996 | Spanish | Cohort, 6 months | PTCA-balloon | diameter stenosis ≥50% | 35/34 | 58.0 | 82.6 |
| Amant [26] | 1997 | French | Cohort, 6 months | PTCA-stent | diameter stenosis >50% | 31/127 | 60.0 | 80.1 |
| Guarda [27] | 1999 | Chilean | Cohort, 6 months | PTCA-stent | diameter stenosis >50% | 22/26 | NA | NA |
| Tsukada [28] | 1997 | Japanese | Cohort, 3 months | PTCA-balloon | diameter stenosis ≥50% | 25/71 | 60.0 | NA |
| Hamon [29] | 1998 | French | Cohort, 6 months | PTCA-balloon | diameter stenosis >50% | 116/155 | 60.0 | 84.5 |
| Gensini [30] | 1999 | Italian | Cohort, 6 months | PTCA-stent | diameter stenosis ≥50% | 27/130 | NA | NA |
| Okamura [31] | 1999 | Japanese | Cohort, 6 months | PTCA-balloon | diameter stenosis >50% | 19/27 | 60.0 | 86.6 |
| Yoshida [32] | 1999 | Japanese | Cohort, 5.21 years | PTCA-balloon | diameter stenosis ≥50% | 47/123 | 58.2 | NA |
| Gürlek [33] | 2000 | Turkish | Cohort, 6 months | PTCA-stent | diameter stenosis ≥50% | 51/107 | 53.0 | 84.8 |
| Völzke [34] | 2000 | German | Cohort, 6 months | PTCA-balloon | diameter stenosis >50% | 160/351 | 60.6 | 75.9 |
| Koch [35] | 2000 | German | Cohort, 1 year | PTCA-stent | diameter stenosis ≥50% | 513/1043 | 62.9 | 78.8 |
| Jørgensen [36] | 2001 | Dane | Cohort, 6 months | PTCA-stent | diameter stenosis >50% | 49/320 | 59.0 | 79.4 |
| Zee [37] | 2001 | Spanish | Cohort, 6 months | PTCA-balloon | diameter stenosis >50% | 342/437 | 58.9 | 89.2 |
| Taniguchi [38] | 2001 | Japanese | Cohort, 6 months | PTCA-stent | diameter stenosis >50% | 26/41 | 65.2 | 74.6 |
| Qu [39] | 2002 | Chinese | Cohort, 3 months | PTCA-stent | diameter stenosis ≥50% | 43/85 | 68.0 | 84.4 |
| Ferrari [40] | 2002 | German | Cohort, 6 months | PTCA-stent | diameter stenosis ≥50% | 39/115 | 61.0 | 77.3 |
| Gomma [41] | 2002 | British | Cohort, 6 months | PTCA-stent | diameter stenosis ≥50% | 60/144 | 59.4 | 75.6 |
| Ryu [42] | 2002 | Korean | Cohort, 6 months | PTCA-stent | diameter stenosis >50% | 64/191 | 59.5 | 74.8 |
| Okumura [43] | 2002 | Japanese | Cohort, 6 months | PTCA-stent | diameter stenosis ≥50% | 16/46 | 64.3 | 79.3 |
| Ribichini [44] | 2003 | Italian | Cohort, 6.3 months | PTCA-stent | diameter stenosis ≥50% | 271/727 | 61.0 | 82.2 |
| Guneri [45] | 2005 | Turkish | Cohort , 9 months | PTCA-stent | diameter stenosis ≥70% | 48/48 | 59.6 | 62.8 |
| Wang [46] | 2005 | Chinese | Cohort, 3 months | PTCA-stent | diameter stenosis ≥50% | 62/40 | 62.0 | 88.2 |
| Wang [47] | 2005 | Chinese | Cohort, 6 months | PTCA-stent | diameter stenosis ≥50% | 58/139 | NA | NA |
| Guo [48] | 2005 | Chinese | Cohort, 6 months | PTCA-stent | diameter stenosis ≥50% | 30/73 | 70.0 | NA |
| Gao [49] | 2006 | Chinese | Cohort, 6 months | PTCA-stent | diameter stenosis ≥50% | 102/247 | NA | NA |
| Wijpkema [50] | 2006 | Dutch | Cohort , 9 months | PTCA-stent | diameter stenosis >50% | 316/2572 | 62.0 | 70.9 |
| Ujiie [51] | 2006 | Japanese | Cohort, 7 months | PTCA-stent | diameter stenosis >50% | 15/60 | 66.9 | 78.6 |
| Lv [52] | 2012 | Chinese | Cohort, 6 months | PTCA-stent | diameter stenosis ≥50% | 81/315 | 58.8 | 89.4 |

NA: not available, FU: follow up
